# Resting-State Functional Connectivity Patterns Predict Acupuncture Treatment Response in Primary Dysmenorrhea

**DOI:** 10.3389/fnins.2020.559191

**Published:** 2020-09-08

**Authors:** Siyi Yu, Mingguo Xie, Shuqin Liu, Xiaoli Guo, Jin Tian, Wei Wei, Qi Zhang, Fang Zeng, Fanrong Liang, Jie Yang

**Affiliations:** ^1^Brain Research Center, Department of Acupuncture & Tuina, Chengdu University of Traditional Chinese Medicine, Chengdu, China; ^2^Department of Radiology, Hospital of Chengdu University of Traditional Chinese Medicine, Chengdu, China

**Keywords:** functional connectivity, primary dysmenorrhea, machine learning, multivariate pattern analyses, acupuncture

## Abstract

Primary dysmenorrhea (PDM) is a common complaint in women throughout the menstrual years. Acupuncture has been shown to be effective in dysmenorrhea; however, there are large interindividual differences in patients’ responses to acupuncture treatment. Fifty-four patients with PDM were recruited and randomized into real or sham acupuncture treatment groups (over the course of three menstrual cycles). Pain-related functional connectivity (FC) matrices were constructed at baseline and post-treatment period. The different neural mechanisms altered by real and sham acupuncture were detected with multivariate analysis of variance. Multivariate pattern analysis (MVPA) based on a machine learning approach was used to explore whether the different FC patterns predicted the acupuncture treatment response in the PDM patients. The results showed that real but not sham acupuncture significantly relieved pain severity in PDM patients. Real and sham acupuncture displayed differences in FC alterations between the descending pain modulatory system (DPMS) and sensorimotor network (SMN), the salience network (SN) and SMN, and the SN and default mode network (DMN). Furthermore, MVPA found that these FC patterns at baseline could predict the acupuncture treatment response in PDM patients. The present study verified differentially altered brain mechanisms underlying real and sham acupuncture in PDM patients and supported the use of neuroimaging biomarkers for individual-based precise acupuncture treatment in patients with PDM.

## Introduction

Primary dysmenorrhea (PDM), cyclic menstrual pain in the absence of pelvic anomalies, is a common, and often debilitating, gynecological condition that affects between 45 and 95% of menstruating women (Coco, 1999). Despite the high incidence rate of PDM, it is unfortunately often underdiagnosed and poorly treated (O’Connell et al., 2006). Acupuncture, a traditional Chinese medicine procedure, has been widely used to alleviate diverse types of pain for over 2000 years (Zhao, 2008). The National Institutes of Health has also recommended acupuncture as an effective tool for certain health problems, including menstrual pain (Campbell and McGrath, 1999). Subsequently, the efficacy and safety of acupuncture for PDM have been reported in our systematic review (Yu et al., 2017) and several randomized controlled trials (RCTs) (Witt et al., 2008; Ma et al., 2013; Armour et al., 2017).

Although acupuncture has been shown to be effective in PDM, patients’ responses to acupuncture treatment vary widely between individuals (Liu et al., 2017; Liu et al., 2019; Tu et al., 2019b). In addition, responses to other analgesic therapies have also been characterized by robust individual differences (Coghill and Eisenach, 2003; Wager et al., 2011; Angst et al., 2012). If we can identify the interindividual differences in pain processing, this would greatly help to achieve improved and personalized treatment. By definition, pain is a subjective and highly personal experience, and treatment outcomes are likely to be affected by an individual’s baseline characteristics, such as demographic characteristics (e.g., sex, race, and age), levels of clinical pain and some objective biological markers (Wandner et al., 2012; Fillingim, 2017). Thus, baseline characteristics of individuals would be useful to predict the differential response to intervention strategies. Previous research has found that baseline clinical and demographic factors influence treatment response, but these characteristics have not achieved the accuracy required for prediction (Underwood et al., 2007; Azevedo et al., 2019; Witt et al., 2019). Some studies have focused on quantitative sensory testing (QST) in the prediction of analgesic effects, but with contradictory results (Grosen et al., 2013). In light of these studies showing limited individual predictive value for clinical measures, brain-based biomarkers have recently shown promise at predicting response to treatment (Chen et al., 2018; Reggente et al., 2018).

PDM has been proposed to be part of the central sensitization syndromes together with several chronic pain conditions, including fibromyalgia, irritable bowel syndrome, idiopathic low back pain, headache and migraine (Yunus, 2012; Iacovides et al., 2015). In recent years, using neuroimaging techniques, our group (Shen et al., 2019; Zhang et al., 2019) and other groups (Tu et al., 2009; Liu et al., 2018; Chen et al., 2019) have confirmed that PDM is associated with significant changes in the central nervous system’s anatomy, metabolism, and resting-state function. Although the exact mechanisms of the analgesic effects of acupuncture are not known, studies have postulated that acupuncture can alleviate pain by modulating brain regions and networks associated with pain processing (Chen et al., 2015; Maeda et al., 2017). Neuroimaging research has demonstrated that chronic pain may be associated with alterations in multiple brain networks, such as the default mode network (DMN), sensorimotor network (SMN), salience network (SN), and descending modulation pathways (DPMS) (Kucyi and Davis, 2015; Kucyi and Davis, 2017). These particular networks, involved in the cognitive, sensorimotor, and affective aspects of pain, have been implicated in the core symptomatology of chronic pain and treatment response (Shi et al., 2015; Wu et al., 2016; Low et al., 2017; Lee et al., 2019; Zhang et al., 2019). However, it remains largely unknown where and how specific changes in these pain-related networks give rise to symptom improvement in patients with PDM after acupuncture treatment and whether network-level markers can predict the clinical response before intervention.

In this study, we used a longitudinal study design to investigate brain plasticity following acupuncture treatment. Our main aim was to explore whether particular pretreatment functional connectivity (FC) patterns (including those of the DMN, SMN, SN and DPMS) would predict the real and sham acupuncture response in PDM patients. First, we assessed FC alterations after acupuncture over the course of three menstrual cycles. Second, the different FC alterations between real and sham acupuncture treatment were explored. Third, we used multivariate pattern analyses (MVPA) based on a machine learning approach (support vector regression, SVR) to explore whether the different FC patterns predicted the acupuncture treatment response in PDM patients. We hypothesized that there were different neural mechanisms underlying real and sham acupuncture treatment that could predict the treatment response in PDM patients.

## Materials and Methods

### Participants

**FIGURE 1:**
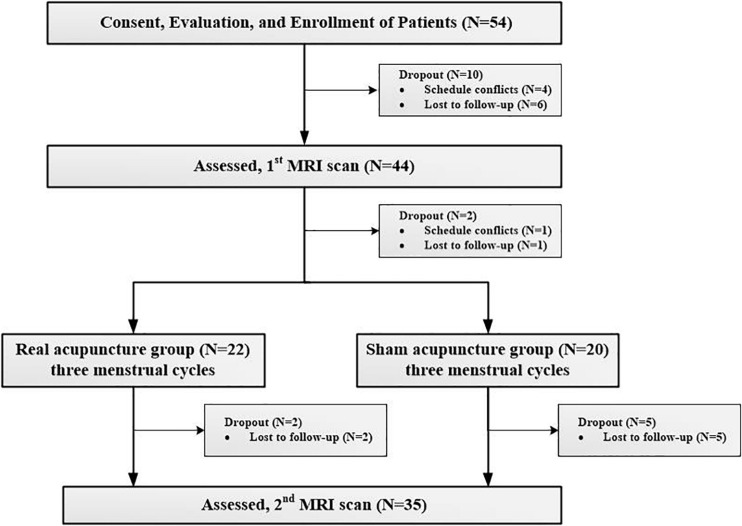
Procedures and data used for the study.

Fifty-four patients with PDM were recruited from advertisements and word of mouth to participate in a dysmenorrhea study, and all participants were screened using telephone and in-person structured interviews. The Research Ethics Committee of Chengdu university of Traditional Chinses Medicine (CDUTCM) approved this study, and all participants gave written informed consent. The inclusion criteria for patients with PDM were (1) a regular menstrual cycle (27–32 days); (2) a history of PDM longer than 1 year; (3) no exogenous hormones or centrally acting medication in the last 6 months; (4) lower quadrant abdominal pain (including cramping, swelling, tingling, etc.) during menstruation in the last 6 months rated higher than 4 on a visual analog scale (VAS) (0 = not at all, 10 = the worst pain sensation); and (5) right-handedness, as confirmed by the Edinburgh Handedness Inventory (Oldfield, 1971). The exclusion criteria for patients with PDM were as follows: (1) other chronic pain conditions, such as low back pain; (2) organic pelvic disease or abnormalities found in gynecological ultrasonography; (3) visceral pain and other neurology that may cause hyperalgesia; (4) a positive pregnancy test or plan for pregnancy; (5) a neurologic or psychiatric disorder history; and (6) any contraindication for MRI scanning. Ten patients dropped out before the baseline clinical assessment and MRI scan, and nine patients dropped out during the treatment period. All the participants did not have any acupuncture experience. Finally, thirty-five patients (20 in the real and 15 in the sham acupuncture groups) completed all the clinical assessments and image scans and received real or sham acupuncture treatment during 3 menstrual cycles (Table 1). The details of the study design can be found in Figure 1.

**TABLE 1:** The demographic and clinical information of each group.

**Items**	**Real acupuncture (*n* = 20)**	**Sham acupuncture (*n* = 15)**	***T***	***P***
Age	24.70(2.10)	24.33(1.84)	0.54	0.59
BMI	19.55(1.38)	19.30(1.69)	0.49	0.63
Duration (months)	91.05(34.88)	91.00(33.72)	0.01	0.99
SAS	41.87(4.77)	40.46(7.82)	0.66	0.51
SDS	40.36(6.61)	43.33(10.17)	0.17	0.30
Baseline VAS	6.17(0.99)	5.87(1.18)	0.94	0.41
Post-treatment VAS	3.35(1.49)	5.39(1.38)	4.03	<0.001
VAS change	3.40(1.53)	1.03(1.84)	4.07	<0.001
VAS change rate (%)	50.80(22.57)	19.42(36.59)	3.49	<0.001

*BMI, body mass index; SAS, self-report anxiety scale; SDS, self-report depression scale; VAS: visual analog scale.*

### Clinical Assessment

The primary outcome assessed in this trial was abdominal pain severity, as measured by the 0–10 VAS rom “no pain at all” to “unbearable pain (Larroy, 2002). In addition, the self-rating anxiety scale (SAS) and self-rating depression scale (SDS) were applied as secondary outcomes to evaluate the anxiety and depression levels of the PDM patients (Zung et al., 1965; Zung, 1971). All clinical outcomes were measured at baseline and after completion of three sessions of treatment during the periovulatory phase (days 12–16 of the menstrual cycle).

### Acupuncture Treatment

Patients were randomized using a computer-generated, random-allocation sequence and then assigned to either the real acupuncture group or the sham acupuncture group. All patients and study staff were blinded to the treatment groups. Only the acupuncturist, who had to know whether to deliver real or sham treatment, was not blinded.

#### Real Acupuncture Treatment

For the real acupuncture treatment group, *sanyinjiao* (SP6) was selected based on data mining from our previous review and expert opinions. SP6 is located on the tibial aspect of the leg, posterior to the medial border of the tibia and 3 *cun* (proportional bone cun), above the medial malleolus (World Health Organization, 2008). The acupuncture procedures were as follows: after the skin was cleaned with tincture of iodine and alcohol, 0.25 × 40 mm stainless needles (Hwatuo, Suzhou, China) were inserted 1.0–1.2 *cun* and gently twisted, lifted and thrust with even amplitude, force and speed four to six times until *deqi* was obtained (soreness, numbness, distension and heaviness). Needles were retained at the acupoints for 30 min, and the above manipulation was repeated twice every 10 min for 30 s each time.

#### Sham Acupuncture Treatment

An adjacent sham acupoint located at the midpoint between the stomach and gall bladder meridians in the same level of SP6 and *xuanzhong* (GB39) was selected in the sham group (Ma et al., 2010). The patients in this group underwent an acupuncture procedure similar to the patients in the real acupuncture group, but no needle manipulation was performed after needle insertion, and *deqi* sensation was not obtained. The real and sham acupuncture targets are displayed in Supplementary Figure S1.

The acupuncture interventions for both groups were performed by two licensed acupuncturists with over 3 years of experience. Acupuncture treatments started 7 days before the beginning of menses and did not stop until the onset of the next menstruation. All participants received acupuncture treatment once a day, there were 7 days in a session, and there were 3 sessions over 3 menstrual cycles.

### Imaging Acquisition

All participants underwent two MRI scans on the same 3.0-Tesla magnetic resonance scanner (Discovery MR750, General Electric, Milwaukee, WI, United States) in the Department of Radiology at the Affiliated Hospital of CDUTCM at baseline and the forth periovulatory phase after each clinical assessment. Tight, but comfortable, foam padding was used to minimize head motion, and earplugs were used to reduce scanner noise. Sagittal 3D T1-weighted images were acquired using a brain volume sequence with the following parameters: repetition time (TR) = 8.16 ms; echo time (TE) = 3.18 ms; flip angle (FA) = 7°; field of view (FOV) = 256 × 256 mm; matrix = 256 × 256; slice thickness = 1 mm, no gap; and 188 sagittal slices. The resting-state functional fMRI (rs-fMRI) datasets were obtained in 7 min with a gradient-recalled echo-planar imaging pulse sequence. The rs-fMRI imaging parameters were TR = 2000 ms, TE = 30 ms, FA = 90°, acquisition matrix = 64 × 64, FOV = 240 × 240 mm, thickness = 4.0 mm, voxel size = 3.5 × 3.5 × 4.02 mm^3^, gap = 0.5 mm, NEX = 1.0, and number of slices = 33. A total of 210 volumes were acquired. All subjects were scanned during the first three days of the menstrual phase. During the data scans, all subjects were instructed to relax and maintain closed eyes, and all participants reported that they did not fall asleep during the scanning.

### fMRI Data Preprocessing

MRI data were preprocessed and analyzed using the SPM8 toolbox^1^ implemented in MATLAB 8.0 (Mathworks Inc., Sherborn, MA, United States). Structural images were coregistered with resting-state functional images. Conventional preprocessing steps were performed, which included (1) removing the first 5 time points; (2) slice timing correction; (3) realignment (participants with head motion greater than 1.5mm maximum displacement in any direction (x, y, z) or 1.5° of angular motion were excluded); (4) normalization of images with a T1 template in the Montreal Neurological Institute (MNI) atlas space and resampling to 3? × 3 × 3mm^3^ cubic voxels; (5) linear detrending; (6) nuisance covariate regression including six motion parameters, average signals of cerebrospinal fluid and white matter, and time points having spike motion of framewise displacement (FD) > 0.5; (7) temporal filtering (bandpass 0.01–0.1 Hz); and (8) spatial smoothing using a Gaussian kernel of 6-mm full-width at half-maximum (FWHM). The images with all preprocessing steps were used for region of interest (ROI) to ROI functional connectivity analysis, while the images after the first four preprocessing steps were used for group independent component analysis (GICA). There were no differences in head motion parameters (FD) within or between groups.

### ROI Selection and Functional Connectivity Analyses

First, GICA analysis was conducted using group ICA of the fMRI toolbox (GIFT 3.0b, Medical Image Analysis Lab, University of New Mexico, Albuquerque, NM, United States) implemented in MATLAB 8.0 and SPM8. A set of independent components (ICs) were identified as intrinsic resting-state networks in all subjects at both the baseline and post-treatment periods (Calhoun et al., 2001). The optimal number of ICs was automatically estimated by minimum description length (MDL) criteria in GIFT, and the median of the MDL over all subjects was 66 (Li et al., 2006). The ICA components were calculated by infomax algorithms, and spatial maps were transformed to z-scores.

**FIGURE 2:**
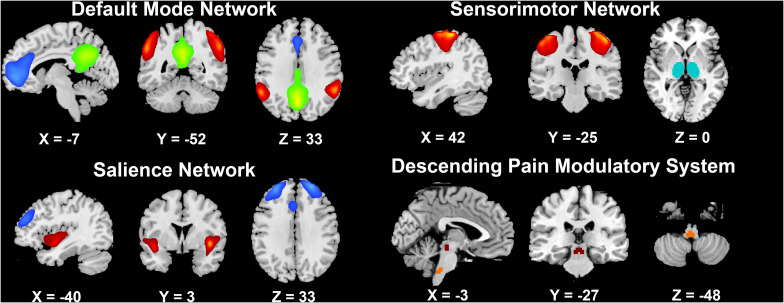
The brain networks selected in the present study.

Second, the default mode network (DMN), sensorimotor network (SMN), and salience network (SN) were chosen as components of interest to be evaluated from the resting-state data. ten ROIs in these resting-state networks (RSNs) were manually chosen from the 66 extracted ICs, which were identified as anatomically and functionally classical RSNs by two experienced neuroimaging researchers (YJ and YSY) (Smith et al., 2009). Four ROIs in the DMN, including the medial prefrontal cortex (mPFC), posterior cingulate cortex (PCC) and bilateral inferior parietal cortices (IPC), are involved in pain rumination (Kumbhare et al., 2017). Four ROIs in the SN, including the bilateral dorsolateral prefrontal cortices (dlPFC) and bilateral anterior insula (aINS) represent the sustained activation during attention to pain (Kucyi and Davis, 2015, 2017), and descending pathways that modulate the transmission of ascending nociceptive signals (Hemington et al., 2016; Davis et al., 2017). Bilateral primary somatosensory cortices (S1) and bilateral thalami in the SMN represent the major ascending pathways of pain (Tracey and Mantyh, 2007; Davis et al., 2017). In addition, two key regions in the descending pain modulatory system, the periaqueductal gray (PAG) and rostroventral medulla (RVM) (Goksan et al., 2018), were also selected as ROIs for the pain-related FC matrix in PDM. The ten cortical ROIs were saved as masks by one sample *t*-test by using SPM8. The subcortical ROI of the thalamus in the SMN was acquired from automated anatomical labeling, and the PAG and RVM masks were acquired from the DPMS network mask in standard space (Goksan et al., 2018; Figure 2).

Third, an ROI-wise FC matrix was constructed using the REST toolbox^2^. The averaged time series in each ROI were extracted, and the Pearson correlations with time series in other ROIs were calculated. Then, the FCs were transformed to *Z* values (Lowe et al., 1998). Thus, a 14 × 14 FC pattern matrix for each individual was separately obtained for further analysis.

### Statistical Analysis

#### Demographic and Clinical Features

Two independent *t*-tests were performed to compare the demographic and clinical traits between the two groups, and paired *t*-tests were employed to determine whether the alterations in VAS scores were significant after treatment in each group (SPSS 20.0; SPSS Inc., Chicago, IL, United States). Pearson correlation analyses were employed to explore associations between duration of disease, baseline VAS, SDS, and SAS scores, and changes of the VAS scores. The significance level was set at *P* < 0.05.

#### Functional Connectivity Difference Analysis

The paired *t*-tests were performed to detect the FC matrix alterations after treatment in each treatment group. In addition, we employed a 2 × 2 (group × time) multivariate analysis of covariance (MANCOVA) on the FC matrix to detect whether there were different neural mechanisms underlying the different treatments, with age and duration of disease as covariates (Hand and Taylor, 1987; Davis, 2002). Traditional Pearson correlation analyses were used to explore the associations between the FCs of MANCOVA and VAS changes after three sessions of acupuncture. The significance level was set at *P* < 0.05 and corrected for multiple comparisons by the false discovery rate (FDR) approach. The in-house script was write in MATLAB to conduct the MANCOVA in the study. The codes for MANCOVA of ROI-wise functional connectivity can be downloaded from https://github.com/cdutcmysy/ROIwise_FC_mancova.

#### MVPA-Based Clinical Symptom Prediction Analysis

MANCOVA found differences in FC between the real and sham treatments for PDM. We proposed that these different FC patterns could reveal the neural mechanisms underlying the effects of acupuncture treatment for PDM and that these FC patterns at baseline would be used for predicting the treatment response to acupuncture in patients with PDM. Here, MVPA based on linear support vector regression (SVR, implemented by LIBSVM^3^) (Chang and Lin, 2011) was employed to verify our hypothesis. We set the change in pain severity (VAS change and VAS change rate) as the dependent variable and FCs from the MANCOVA results at baseline as independent variables (predictors) in all participants and regressed out the effects of age, treatment method and duration of disease. A leave-one-out cross-validation (LOOCV) method was used for prediction to ensure separation between training and testing samples (Plitt et al., 2015). We calculated the squared prediction-outcome correlation (*R*^2^) as well as the mean absolute error (MAE) to evaluate the SVR predictive ability (Wager et al., 2013; Lindquist et al., 2017). Furthermore, we employed the permutation test to verify that the predictor was not from a random chance (repeated 5000 times).

## Results

### Demographic and Clinical Features

There were no significant differences in age, BMI, duration of disease, and baseline SAS, SDS or VAS scores between the real and sham acupuncture treatment groups (all *P* > 0.05; see Table 1). The VAS at the post-treatment period was lower in the real group than in the sham group, and the VAS change score and VAS change rate were significantly higher in the real acupuncture treatment group than in the sham acupuncture treatment group. Paired *t*-tests showed that the VAS change in the real acupuncture treatment group was significant (*t* = 9.90, *P* < 0.01), while the VAS change in the sham acupuncture group was not significant (*t* = 2.10, *P* = 0.06). In addition, there were no significant associations between duration of disease and baseline VAS, SDS, and SAS scores and post-treatment VAS score changes in either group (all *P* > 0.05; see Table 2).

**TABLE 2:** The correlations between baseline clinical features, FCs and VAS changes in PDM patients.

**Items**	**VAS change**		**VAS change rate**	
	***R***	***P***	***R***	***P***
Duration of disease	0.05	0.80	0.11	0.54
Baseline VAS score	−0.15	0.39	0.05	0.78
Baseline SDS score	−0.19	0.29	−0.23	0.19
Baseline SAS score	−0.00	0.98	−0.11	0.52
L_S1-R_aINs	0.05	0.81	0.00	0.99
R_S1-R_aINs	−0.10	0.60	−0.10	0.59
L_S1-R_dlPFC	0.09	0.64	0.05	0.79
R_S1-R_dlPFC	−0.01	0.95	−0.15	0.42
RVM-L_Tha	0.15	0.42	0.33	0.07
RVM-R_Tha	0.23	0.21	0.21	0.25
RVM-PAG	−0.17	0.37	−0.24	0.20
L_IPC-R_dlPFC	0.02	0.93	0.17	0.37

*FC, functional connectivity; VAS: visual analog scale; PDM, primary dysmenorrhea; SAS, self-report anxiety scale; SDS, self-report depression scale; L, left side; R, right side; S1, primary somatosensory cortex; aINS, anterior insula; dlPFC, dorsolateral prefrontal cortex; RVM, rostroventral medulla; Tha, thalamus; PAG, periaqueductal gray; IPC, inferior parietal cortex.*

### FC Matrix Alterations After Real and Sham Acupuncture Treatment

**FIGURE 3:**
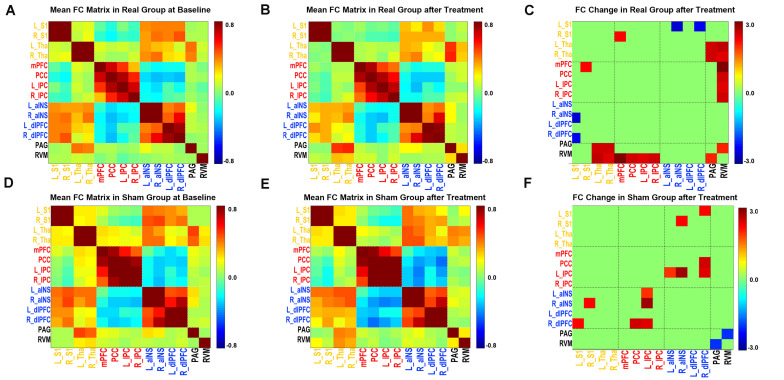
FC matrix pattern in each group. **(A,D)** The FC matrix pattern at baseline. **(B,E)** The FC matrix pattern after treatment. The color bar indicates the correlation coefficient between two regions. **(C,F)** FC alterations after acupuncture treatment; the color bar indicates the *T* value. **(A,B,C)** Real acupuncture group. **(D,E,F)** Sham acupuncture group. Abbreviations: FC, functional connectivity; L, left side; R, right side; S1, primary somatosensory cortex; Tha, thalamus; mPFC, medial prefrontal cortex; PCC, posterior cingulate cortex; IPC, inferior parietal cortex; aINS, anterior insula; dlPFC, dorsolateral prefrontal cortex; RVM, rostroventral medulla; PAG, periaqueductal gray.

As illustrated in Figures 3A–C, after real acupuncture treatment, both increased and decreased FCs were found; specifically, FCs between the mPFC-right S1, PAG-bilateral thalami, PAG-RVM, RVM-bilateral thalami and RVM-DMN (all four ROIs in the DMN) were increased, while FC between the left S1-right aINS was decreased after real acupuncture treatment. For the sham acupuncture treatment group, increased FCs were found in the right S1-right aINS, left IPC-bilateral aINS, right dlPFC-left S1, right dlPFC-PCC and right dlPFC-right IPC, while FC between the PAG-RVM was decreased after sham treatment (Figures 3D–F). Interestingly, we did not find the same patterns of altered FC in the real and sham acupuncture treatment groups.

### Group Differences in the FC Matrix

**FIGURE 4:**
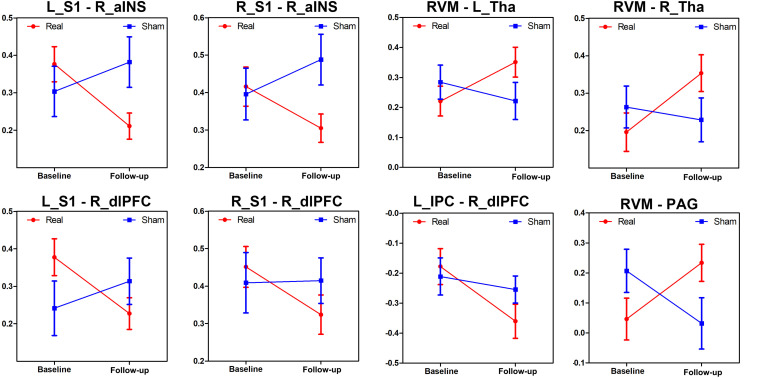
Changes in the different brain connections following real and sham acupuncture for PDM. The line charts display the different alterations between real and sham acupuncture for PDM in these FCs after treatment. Abbreviations: FC, functional connectivity; L, left side; R, right side; S1, primary somatosensory cortex; aINS, anterior insula; dlPFC, dorsolateral prefrontal cortex; RVM, rostroventral medulla; Tha, thalamus; PAG, periaqueductal gray; IPC, inferior parietal cortex.

The MANCOVA analysis results are displayed in Figure 4 and Supplementary Figure S2. Eight paired FCs showed significant differences between the two groups, including the right aINS-bilateral S1, right dlPFC-bilateral S1, right dlPFC-left IPC, RVM-bilateral thalami and RVM-PAG. Specifically, FCs that were decreased in the real group but increased in the sham group were located in the SMN-SN and DMN-SN, while FCs that were increased in the real group but decreased in the sham group were found in the DPMS-SMN and within the DPMS pathway.

### Clinical Prediction Results

**FIGURE 5:**
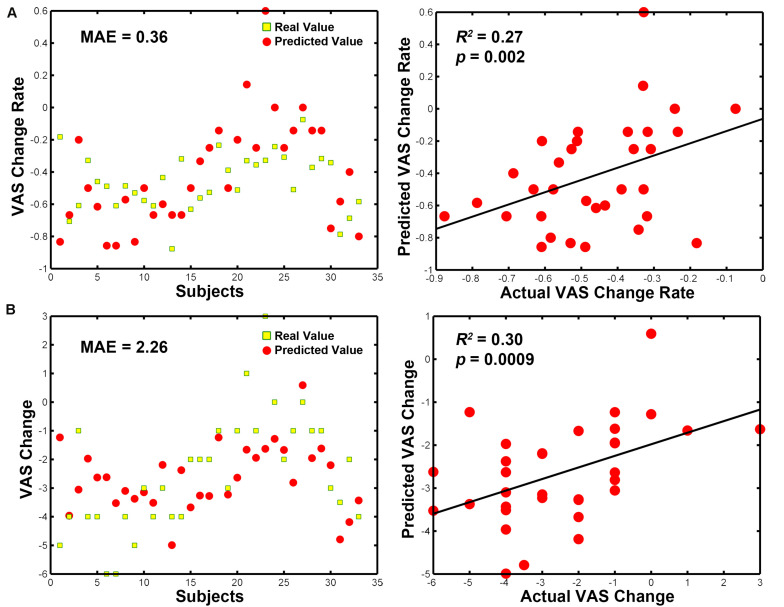
MVPA-based clinical prediction results. **(A)** Baseline FC patterns predict VAS change scores after treatment. **(B)** Baseline FC patterns predict VAS change rates after treatment. Abbreviations: MVPA, multivariate pattern analysis; MAE, mean absolute error; VAS, visual analog scale.

As we found significant group differences in FC alterations between the real and sham groups, we used the baseline FCs as predictors of the treatment response and controlled for the effect of age, duration and treatment method. The SVR analyses revealed that the eight baseline FC patterns predicted the VAS change scores (*R*^2^ = 0.27, *P* = 0.002, MAE = 0.36; Figure 5A) and VAS change rate after treatment (*R*^2^ = 0.30, *P* = 0.0009, MAE = 2.26; Figure 5B). The permutation tests confirmed that the results could not be obtained by chance (*P* < 0.001). Conversely, the traditional bivariate correlation analyses did not find any significant associations between these differences in FC and VAS changes after acupuncture treatment in the patients with PDM (all *P* > 0.05; see Table 2).

## Discussion

This study in patients with PDM used MVPA recognition to identify neurobiological predictors of acupuncture treatment response. We first verified the efficacy of real but not sham acupuncture treatment in the patients with PDM. Then, we identified FCs involving the SMN-SN, DMN-SN, DPMS-SMN and DPMS pathway that showed different alterations after real and sham acupuncture treatment in PDM patients. Furthermore, MVPA revealed that these pretreatment multivariate FC patterns in the SMN, SN, DMN and DPMS significantly predicted individual patient pain symptoms after 3 menstrual cycles of intensive acupuncture treatment. Conversely, pretreatment clinical variables and traditional correlation analyses were not able to predict posttreatment pain severity in these patients with PDM. These findings have implications for identifying who will benefit most from acupuncture, as well as for understanding the pathophysiology of PDM as it relates to acupuncture effects.

The present study verified the effect of acupuncture on reducing menstrual pain intensity in PDM (Yu et al., 2017). In the present study, for the real acupuncture treatment, we selected *sanyinjiao* (SP6) as the acupuncture target based on data mining from our previous review (Yu et al., 2015) and expert opinions, while for the sham acupuncture treatment, an adjacent non-acupoint was selected. We found that real acupuncture was superior to sham acupuncture for pain relief. The results indicated that correct acupuncture point locations are important contributors to the treatment effects and that the acupuncture effect of pain relief cannot be explained solely in terms of placebo effects (Smith et al., 2011; Vickers et al., 2018). Subsequently, we detected the potential brain mechanisms underlying the real and sham acupuncture treatment effects in PDM.

To date, no study has explored the underlying mechanisms of pain reduction by acupuncture in patients with PDM. For other types of chronic pain, Lee et al. found that both real and sham (phantom) acupuncture could reduce pain ratings in patients with low back pain (LBP), but the reduced pain intensity was associated with reduced FC between the mPFC and posterior insula in the real acupuncture group, while the decreased pain intensity was associated with enhanced FC between the PCC and aINS (Lee et al., 2019). The results indicated differential brain influences on real and sham acupuncture in LBP. Here, we constructed the pain-related functional connectivity matrices in patients with PDM by combining the data-driven approach (GICA) and hypothesis-based ROI selection method with rs-fMRI data. The findings showed that altered pain-related FCs after real acupuncture were located in FC between the DPMS-SMN and DPMS-DMN and within the DPMS, while altered FCs after sham acupuncture were found in FC between the SN-SMN and SN-DMN and within the DPMS. In general, real acupuncture modulated the DPMS-associated brain network more, while sham acupuncture modulated the SN-related brain network more. These results indicated that real acupuncture could modulate widespread brain networks associated with the DPMS, while sham acupuncture had a more focused modulation in the salience network associated with analgesia in patients with PDM. Furthermore, MANCOVA was used to detect the different brain functional modulatory mechanisms between real and sham acupuncture treatment in patients with PDM.

We found eight functional couplings that showed differences in alterations after real and sham treatment of PDM. As illustrated in Figure 3, real acupuncture increased but sham acupuncture decreased FC between the DPMS and SMN and FC within the DPMS (RVM-PAG), while real acupuncture reduced but sham acupuncture enhanced FC between the SN and SMN, and the SN and DMN. Our study was consistent with previous findings of different modulatory effects in the central nervous system between real and sham acupuncture in a healthy population. Cao et al. found that real acupuncture could increase pain intensity thresholds, which were associated with increased neural activity in the insula (Cao et al., 2018). A recent neuroimaging meta-analysis about acupuncture effects in the brain in healthy individuals found that in 29 out of 33 studies, real acupuncture, compared to sham acupuncture, resulted in more/different modulatory effects on neurological components measured by neuroimaging, including somatosensory, affective, and cognitive aspects (Scheffold et al., 2015). The dlPFC is known to be associated with center control and top-down processes for pain control (Wager et al., 2004; Kong et al., 2006), as well as anticipation of pain relief and expectation-related placebo analgesia (Krummenacher et al., 2010). The SMN represents the major ascending pathways of pain (Tracey and Mantyh, 2007; Davis et al., 2017), while the DMN is involved in the self-regulation of pain, such as mind wandering (Kucyi et al., 2013; Kucyi and Davis, 2015). Multiple neuroimaging studies have suggested that acupuncture may achieve analgesic effects by modulating the SN, DMN and SMN (Chen et al., 2015; Shi et al., 2015; Lee et al., 2019; Zhang et al., 2019). The DPMS is considered the key system for pain modulation (Millan, 2002) and has also been found to be modulated by acupuncture treatment in migraine (Li et al., 2016). The different modulatory effects of real and sham acupuncture in the pain-associated brain networks indicated different and complex neural mechanisms of real and sham acupuncture in patients with PDM. The different alterations in these pain-related FCs resulted in different treatment responses, which also indicated the potential use of these FCs as predictors for acupuncture treatment response in PDM patients.

Our research is in line with the growing interest in predictive modeling using neuroimaging and machine learning methods (Janssen et al., 2018; Keshavan et al., 2020). Most machine learning studies have focused on differential diagnosis, identifying brain signatures that discriminate patients from healthy controls, and further established objective signs of disease pathology (Lai, 2019). Another important application of machine learning has been the use of brain characteristics to predict therapeutic outcomes and offer personalized tailoring of interventions (Gao et al., 2018; Leaver et al., 2018; Mithani et al., 2019). Here, using MVPA (Yang et al., 2012), a widely applied machine learning approach, we found that several pain-related FC patterns at baseline could be a useful predictor for acupuncture treatment response (both VAS changes and the change rate) in PDM patients. Recently, Tu et al. also used MVPA-based rs-FCs to predict real and sham acupuncture treatment responses in chronic LBP, and the rs-FC characteristics were significantly predictive of the differential responses to real and sham treatment in LBP (Tu et al., 2019a). In our study, the predictive power and strength of the MVPA approach was validated in several ways. First, we found that baseline clinical or demographic features were unable to predict the outcome responses of the PDM patients. Second, the traditional univariate correlations between FCs and VAS changes were not significant. Given the nature of MVPA approaches, these techniques provide improved sensitivity to subtle and spatially distributed brain differences that would likely remain undetected with the use of conventional univariate approaches (Fan et al., 2006; Haxby, 2012). Therefore, we demonstrated the feasibility and reliability of the MVPA model for predicting clinical symptom changes after acupuncture treatment in patients with PDM.

## Limitations

The present study also has several limitations. First, the sample size in this study was small, especially in the sham acupuncture group, and previous studies have also found a significant analgesic effect of sham acupuncture (Tu et al., 2019a). Our study found that the VAS change in the sham acupuncture group was nearly significant (*P* = 0.06), and further studies with larger sample sizes are needed to address this point. Second, as the PDM is a cyclic chronic pain, the present study only explored the “trait pain” (average pain experienced over time) in the patients with PDM, and future studies should also explore the mechanism of the acupuncture-related analgesic effect on “state pain” (pain at the menstrual period) in PDM patients (Low et al., 2018). Third, to increase the statistical power, we combined the two treatment groups as a pooled group in the MVPA analysis after controlling for the effect of treatment. Nevertheless, we did not find that baseline FCs predicted the treatment response in the single groups. Further studies with larger sample sizes are needed to further detect the different predictors for real and sham treatment effects in PDM.

## Conclusion

The present study verified the different brain mechanisms underlying real and sham acupuncture in PDM patients. In addition, the pain-related FC patterns at baseline could predict the acupuncture treatment response for patients with PDM. The findings supported the use of neuroimaging biomarkers for individual-based precise acupuncture treatment in patients with PDM.

## Data Availability Statement

The raw data supporting the conclusions of this article will be made available by the authors, without undue reservation.

## Ethics Statement

The studies involving human participants were reviewed and approved by the Research Ethics Committee of Chengdu University of Traditional Chinses Medicine. The patients/participants provided their written informed consent to participate in this study.

## Author Contributions

JY and FL designed the study. SL, WW, XG, JT, QZ, and MX collected the data. SY and MX analyzed the data. SY contributed to the original draft. JY, FZ, and FL reviewed and edited the manuscript. All authors contributed to the article and approved the submitted version.

## Conflict of Interest

The authors declare that the research was conducted in the absence of any commercial or financial relationships that could be construed as a potential conflict of interest.

## Footnotes

1 http://www.fil.ion.ucl.ac.uk/spm

2 http://www.restfmri.net

3 https://www.csie.ntu.edu.tw/∼cjlin/libsvm/

## References

[B1] Angst M. S. Phillips N. G. Drover D. R. Tingle M. Ray A. Swan G. E. (2012). Pain sensitivity and opioid analgesia: a pharmacogenomic twin study. *Pain* 153 1397–1409. 10.1016/j.pain.2012.02.022 22444188 PMC3377769

[B2] Armour M. Dahlen H. G. Zhu X. Farquhar C. Smith C. A. (2017). The role of treatment timing and mode of stimulation in the treatment of primary dysmenorrhea with acupuncture: an exploratory randomised controlled trial. *PLoS One* 12:e0180177. 10.1371/journal.pone.0180177 28700680 PMC5507497

[B3] Azevedo D. C. Ferreira P. H. Santos H. O. Oliveira D. R. Souza J. V. L. Costa L. O. P. (2019). Baseline characteristics did not identify people with low back pain who respond best to a movement system impairment-based classification treatment. *Braz. J. Phys. Ther.* 24 358–364. 31230988 10.1016/j.bjpt.2019.05.006 PMC7352034

[B4] Calhoun V. D. Adali T. Pearlson G. D. Pekar J. J. (2001). A method for making group inferences from functional MRI data using independent component analysis. *Hum. Brain Mapp.* 14 140–151. 10.1002/hbm.1048 11559959 PMC6871952

[B5] Campbell M. A. McGrath P. J. (1999). Non-pharmacologic strategies used by adolescents for the management of menstrual discomfort. *Clin. J. Pain* 15 313–320. 10.1097/00002508-199912000-00008 10617260

[B6] Cao J. Tu Y. Orr S. P. Lang C. Park J. Vangel M. (2018). Analgesic effects evoked by real and imagined acupuncture: a neuroimaging study. *Cereb. Cortex* 29 3220–3231. 10.1093/cercor/bhy190 30137262 PMC7302519

[B7] Chang C. C. Lin C. J. (2011). LIBSVM: a library for support vector machines. *ACM Trans. Intell. Syst. Tech.* 2 1–27. 10.1145/1961189.1961199

[B8] Chen J. Wang Z. Tu Y. Liu X. Jorgenson K. Ye G. (2018). Regional homogeneity and multivariate pattern analysis of cervical spondylosis neck pain and the modulation effect of treatment. *Front. Neurosci.* 12:900. 10.3389/fnins.2018.00900 30574062 PMC6292425

[B9] Chen T. Mu J. Xue Q. Yang L. Dun W. Zhang M. (2019). Whole-brain structural magnetic resonance imaging–based classification of primary dysmenorrhea in pain-free phase: a machine learning study. *Pain* 160 734–741. 10.1097/j.pain.0000000000001428 30376532

[B10] Chen X. Spaeth R. B. Freeman S. G. Scarborough D. M. Hashmi J. A. Wey H.-Y. (2015). The modulation effect of longitudinal acupuncture on resting state functional connectivity in knee osteoarthritis patients. *Mol. Pain* 11:67. 10.1186/s12990-015-0071-9 PMC4625557 26511911

[B11] Coco A. S. (1999). Primary dysmenorrhea. *Am. Family Phys.* 60 489–496. 10465224

[B12] Coghill R. C. Eisenach J. (2003). Individual differences in pain sensitivity: implications for treatment decisions. *Anesthesiology* 98 1312–1314. 10.1097/00000542-200306000-00003 12766637

[B13] Davis C. S. (2002). Normal-theory methods: multivariate analysis of variance. *Statist. Methods Anal. Repeat. Meas.* 133 73–102. 10.1007/978-0-387-21573-0_4

[B14] Davis K. D. Flor H. Greely H. T. Iannetti G. D. Mackey S. Ploner M. (2017). Brain imaging tests for chronic pain: medical, legal and ethical issues and recommendations. *Nat. Rev. Neurol.* 13:624. 10.1038/nrneurol.2017.122 28884750

[B15] Fan Y. Shen D. Gur R. C. Gur R. E. Davatzikos C. (2006). COMPARE: classification of morphological patterns using adaptive regional elements. *IEEE Trans. Med. Imaging* 26 93–105. 10.1109/tmi.2006.886812 17243588

[B16] Fillingim R. B. (2017). Individual differences in pain: understanding the mosaic that makes pain personal. *Pain* 158:S11. 10.1097/j.pain.0000000000000775 PMC5350021 27902569

[B17] Gao S. Calhoun V. D. Sui J. (2018). Machine learning in major depression: from classification to treatment outcome prediction. *CNS Neurosci. Ther.* 24 1037–1052. 10.1111/cns.13048 30136381 PMC6324186

[B18] Goksan S. Baxter L. Moultrie F. Duff E. Hathway G. Hartley C. (2018). The influence of the descending pain modulatory system on infant pain-related brain activity. *eLife* 7:e37125. 10.7554/eLife.37125 PMC6133549 30201093

[B19] Grosen K. Fischer I. W. D. Olesen A. E. Drewes A. (2013). Can quantitative sensory testing predict responses to analgesic treatment? *Eur. J. Pain* 17 1267–1280. 10.1002/j.1532-2149.2013.00330.x 23658120

[B20] Hand D. J. Taylor C. C. (1987). *Multivariate Analysis of Variance and Repeated Measures: A Practical Approach for Behavioral Scientists.* London: CRC press.

[B21] Haxby J. V. (2012). Multivariate pattern analysis of fMRI: the early beginnings. *Neuroimage* 62 852–855. 10.1016/j.neuroimage.2012.03.016 22425670 PMC3389290

[B22] Hemington K. S. Wu Q. Kucyi A. Inman R. D. Davis K. D. (2016). Abnormal cross-network functional connectivity in chronic pain and its association with clinical symptoms. *Brain Struct. Funct.* 221 4203–4219. 10.1007/s00429-015-1161-1 26669874

[B23] Iacovides S. Avidon I. Baker F. C. (2015). What we know about primary dysmenorrhea today: a critical review. *Hum. Reprod. Update* 21 762–778. 10.1093/humupd/dmv039 26346058

[B24] Janssen R. J. Mourão-Miranda J. Schnack H. G. (2018). Making individual prognoses in psychiatry using neuroimaging and machine learning. *Biol. Psychiatry* 3 798–808. 10.1016/j.bpsc.2018.04.004 29789268

[B25] Keshavan M. S. Collin G. Guimond S. Kelly S. Prasad K. M. Lizano P. (2020). Neuroimaging in Schizophrenia. *Neuroimaging Clin. N. Am.* 30 73–83. 31759574 10.1016/j.nic.2019.09.007 PMC7724147

[B26] Kong J. Gollub R. L. Rosman I. S. Webb J. M. Vangel M. G. Kirsch I. (2006). Brain activity associated with expectancy-enhanced placebo analgesia as measured by functional magnetic resonance imaging. *J. Neurosci.* 26 381–388. 10.1523/jneurosci.3556-05.2006 16407533 PMC6674420

[B27] Krummenacher P. Candia V. Folkers G. Schedlowski M. Schönbächler G. (2010). Prefrontal cortex modulates placebo analgesia. *Pain* 148 368–374. 10.1016/j.pain.2009.09.033 19875233

[B28] Kucyi A. Davis K. D. (2015). The dynamic pain connectome. *Trends Neurosci.* 38 86–95. 10.1016/j.tins.2014.11.006 25541287

[B29] Kucyi A. Davis K. D. (2017). The neural code for pain: from single-cell electrophysiology to the dynamic pain connectome. *Neuroscientist* 23 397–414. 10.1177/1073858416667716 27660241

[B30] Kucyi A. Salomons T. V. Davis K. D. (2013). Mind wandering away from pain dynamically engages antinociceptive and default mode brain networks. *Proc. Natl. Acad. Sci. U.S.A.* 110 18692–18697. 10.1073/pnas.1312902110 24167282 PMC3832014

[B31] Kumbhare D. A. Elzibak A. H. Noseworthy M. D. (2017). Evaluation of chronic pain using magnetic resonance (MR) neuroimaging approaches. *Clin. J. Pain* 33 281–290. 10.1097/ajp.0000000000000415 27518493

[B32] Lai C. H. (2019). The neural markers of MRI to differentiate depression and panic disorder. *Prog. Neuropsychopharmacol. Biol. Psychiatry* 91 72–78. 10.1016/j.pnpbp.2018.04.013 29705713

[B33] Larroy C. (2002). Comparing visual-analog and numeric scales for assessing menstrual pain. *Behav. Med.* 27 179–181. 10.1080/08964280209596043 12165972

[B34] Leaver A. M. Wade B. Vasavada M. Hellemann G. Joshi S. H. Espinoza R. (2018). Fronto-temporal connectivity predicts ECT outcome in major depression. *Front. Psychiatry* 9:92. 10.3389/fpsyt.2018.00092 29618992 PMC5871748

[B35] Lee J. Eun S. Kim J. Lee J.-H. Park K. (2019). Differential influence of acupuncture somatosensory and cognitive/affective components on functional brain connectivity and pain reduction during low back pain state. *Front. Neurosci.* 13:1062. 10.3389/fnins.2019.01062 31636536 PMC6788296

[B36] Li Y. O. Adali T. Calhoun V. D. (2006). “Sample dependence correction for order selection in fMRI analysis,” in *Proceedings of the IEEE International Symposium on Biomedical Imaging: Nano To Macro*, (Arlington, VA: IEEE), 1072–1075.

[B37] Li Z. Liu M. Lan L. Zeng F. Makris N. Liang Y. (2016). Altered periaqueductal gray resting state functional connectivity in migraine and the modulation effect of treatment. *Sci. Rep.* 6:20298. 10.1038/srep20298 PMC4738255 26839078

[B38] Lindquist M. A. Krishnan A. Lopez-Sola M. Jepma M. Woo C. W. Koban L. (2017). Group-regularized individual prediction: theory and application to pain. *Neuroimage* 145 274–287. 10.1016/j.neuroimage.2015.10.074 26592808 PMC5071107

[B39] Liu J. Mu J. Chen T. Zhang M. Tian J. (2019). White matter tract microstructure of the mPFC-amygdala predicts interindividual differences in placebo response related to treatment in migraine patients. *Hum. Brain Mapp.* 40 284–292. 10.1002/hbm.24372 30256491 PMC6865394

[B40] Liu J. Mu J. Liu Q. Dun W. Zhang M. Tian J. (2017). Brain structural properties predict psychologically mediated hypoalgesia in an 8-week sham acupuncture treatment for migraine. *Hum. Brain Mapp.* 38 4386–4397. 10.1002/hbm.23667 28608601 PMC6866832

[B41] Liu P. Liu Y. Wang G. Li R. Wei Y. Fan Y. (2018). Changes of functional connectivity of the anterior cingulate cortex in women with primary dysmenorrhea. *Brain Imaging Behav.* 12 710–717. 10.1007/s11682-017-9730-y 28516336

[B42] Low I. Kuo P.-C. Liu Y.-H. Tsai C.-L. Chao H.-T. Hsieh J.-C. (2017). Altered brain complexity in women with primary dysmenorrhea: a resting-state magneto-encephalography study using multiscale entropy analysis. *Entropy* 19:680 10.3390/e19120680

[B43] Low I. Wei S.-Y. Lee P.-S. Li W.-C. Lee L.-C. Hsieh J.-C. (2018). Neuroimaging studies of primary Dysmenorrhea. *Adv. Exp. Med.Biol.* 1099 179–199. 30306525 10.1007/978-981-13-1756-9_16

[B44] Lowe M. J. Mock B. J. Sorenson J. A. (1998). Functional connectivity in single and multislice echoplanar imaging using resting-state fluctuations. *Neuroimage* 7 119–132. 10.1006/nimg.1997.0315 9558644

[B45] Ma Y.-X. Ma L.-X. Liu X.-L. Ma Y.-X. Lv K. Wang D. (2010). A comparative study on the immediate effects of electroacupuncture at Sanyinjiao (SP6), Xuanzhong (GB39) and a non-meridian point, on menstrual pain and uterine arterial blood flow, in primary dysmenorrhea patients. *Pain Med.* 11 1564–1575. 10.1111/j.1526-4637.2010.00949.x 21199306

[B46] Ma Y. X. Ye X. N. Liu C. Z. Cai P. Y. Li Z. F. Du D. Q. (2013). A clinical trial of acupuncture about time-varying treatment and points selection in primary dysmenorrhea. *J. Ethnopharmacol.* 148 498–504. 10.1016/j.jep.2013.04.045 23684618

[B47] Maeda Y. Kim H. Kettner N. Kim J. Cina S. Malatesta C. (2017). Rewiring the primary somatosensory cortex in carpal tunnel syndrome with acupuncture. *Brain* 140 914–927. 10.1093/brain/awx015 28334999 PMC5837382

[B48] Millan M. J. (2002). Descending control of pain. *Prog. Neurobiol.* 66 355–474. 12034378 10.1016/s0301-0082(02)00009-6

[B49] Mithani K. Mikhail M. Morgan B. R. Wong S. Weil A. G. Deschenes S. (2019). Connectomic profiling identifies responders to vagus nerve stimulation. *Ann. Neurol.* 86 743–753. 10.1002/ana.25574 31393626

[B50] O’Connell K. Davis A. R. Westhoff C. (2006). Self-treatment patterns among adolescent girls with dysmenorrhea. *J. Pediatr Adolescent Gynecol.* 19 285–289. 10.1016/j.jpag.2006.05.004 16873033

[B51] Oldfield R. C. (1971). The assessment and analysis of handedness: the Edinburgh inventory. *Neuropsychologia* 9 97–113. 10.1016/0028-3932(71)90067-4 5146491

[B52] Plitt M. Barnes K. A. Wallace G. L. Kenworthy L. Martin A. (2015). Resting-state functional connectivity predicts longitudinal change in autistic traits and adaptive functioning in autism. *Proc. Natl. Acad. Sci. U.S.A.* 112 E6699–E6706. 26627261 10.1073/pnas.1510098112 PMC4672806

[B53] Reggente N. Moody T. D. Morfini F. Sheen C. Rissman J. O’neill J. (2018). Multivariate resting-state functional connectivity predicts response to cognitive behavioral therapy in obsessive-compulsive disorder. *Proc. Natl. Acad. Sci. U.S.A.* 115 2222–2227. 10.1073/pnas.1716686115 29440404 PMC5834692

[B54] Scheffold B. E. Hsieh C.-L. Litscher G. (2015). Neuroimaging and neuromonitoring effects of electro and manual acupuncture on the central nervous system: a literature review and analysis. *Evid. Based Comp. Alternat. Med.* 2015:641742. 10.1155/2015/641742 PMC4538975 26339269

[B55] Shen Z. Yu S. Wang M. She T. Yang Y. Wang Y. (2019). Abnormal amygdala resting-state functional connectivity in primary dysmenorrhea. *NeuroReport* 30 363–368. 10.1097/wnr.0000000000001208 30762615

[B56] Shi Y. Liu Z. Zhang S. Li Q. Guo S. Yang J. (2015). Brain network response to acupuncture stimuli in experimental acute low back pain: an fMRI study. *Evid. Based Comp.Alternat. Med.* 2015:210120. 10.1155/2015/210120 PMC4487721 26161117

[B57] Smith C. A. Zhu X. He L. Song J. (2011). Acupuncture for primary dysmenorrhoea. *Cochrane Database Syst. Rev.* 9:CD007854. 10.1002/14651858.CD007854.pub2 21249697

[B58] Smith S. M. Fox P. T. Miller K. L. Glahn D. C. Fox P. M. Mackay C. E. (2009). Correspondence of the brain’s functional architecture during activation and rest. *Proc. Natl. Acad. Sci. U.S.A.* 106 13040–13045. 19620724 10.1073/pnas.0905267106 PMC2722273

[B59] Tracey I. Mantyh P. W. (2007). The cerebral signature for pain perception and its modulation. *Neuron* 55 377–391. 10.1016/j.neuron.2007.07.012 17678852

[B60] Tu C.-H. Niddam D. M. Chao H.-T. Liu R.-S. Hwang R.-J. Yeh T.-C. (2009). Abnormal cerebral metabolism during menstrual pain in primary dysmenorrhea. *Neuroimage* 47 28–35. 10.1016/j.neuroimage.2009.03.080 19362153

[B61] Tu Y. Jung M. Gollub R. L. Napadow V. Gerber J. Ortiz A. (2019a). Abnormal medial prefrontal cortex functional connectivity and its association with clinical symptoms in chronic low back pain. *Pain* 160 1308–1318. 10.1097/j.pain.0000000000001507 31107712 PMC6530583

[B62] Tu Y. Ortiz A. Gollub R. L. Cao J. Gerber J. Lang C. (2019b). Multivariate resting-state functional connectivity predicts responses to real and sham acupuncture treatment in chronic low back pain. *Neuroimage Clin.* 23:101885. 10.1016/j.nicl.2019.101885 31176295 PMC6551557

[B63] Underwood M. Morton V. Farrin A. Team U. B. T. (2007). Do baseline characteristics predict response to treatment for low back pain? Secondary analysis of the UK BEAM dataset [ISRCTN32683578]. *Rheumatology* 46 1297–1302. 10.1093/rheumatology/kem113 17522096

[B64] Vickers A. J. Vertosick E. A. Lewith G. Macpherson H. Foster N. E. Sherman K. J. (2018). Acupuncture for chronic pain: update of an individual patient data meta-analysis. *J. Pain* 19 455–474. 10.1016/j.jpain.2017.11.005 29198932 PMC5927830

[B65] Wager T. D. Atlas L. Y. Leotti L. A. Rilling J. K. (2011). Predicting individual differences in placebo analgesia: contributions of brain activity during anticipation and pain experience. *J. Neurosci.* 31 439–452. 10.1523/jneurosci.3420-10.2011 21228154 PMC3735131

[B66] Wager T. D. Atlas L. Y. Lindquist M. A. Roy M. Woo C. W. Kross E. (2013). An fMRI-based neurologic signature of physical pain. *N. Engl. J. Med.* 368 1388–1397. 10.1056/nejmoa1204471 23574118 PMC3691100

[B67] Wager T. D. Rilling J. K. Smith E. E. Sokolik A. Casey K. L. Davidson R. J. (2004). Placebo-induced changes in FMRI in the anticipation and experience of pain. *Science* 303 1162–1167. 10.1126/science.1093065 14976306

[B68] Wandner L. D. Scipio C. D. Hirsh A. T. Torres C. A. Robinson M. E. (2012). The perception of pain in others: how gender, race, and age influence pain expectations. *J. Pain* 13 220–227. 10.1016/j.jpain.2011.10.014 22225969 PMC3294006

[B69] Witt C. M. Reinhold T. Brinkhaus B. Roll S. Jena S. Willich S. N. (2008). Acupuncture in patients with dysmenorrhea: a randomized study on clinical effectiveness and cost-effectiveness in usual care. *Am. J. Obstet. Gynecol.* 198 166.e1–166.e8. 18226614 10.1016/j.ajog.2007.07.041

[B70] Witt C. M. Vertosick E. A. Foster N. E. Lewith G. Linde K. Macpherson H. (2019). The effect of patient characteristics on acupuncture treatment outcomes: an individual patient data meta-analysis of 20,827 chronic pain patients in randomized controlled trials. *Clin. J. Pain* 35 428–434. 10.1097/ajp.0000000000000691 30908336 PMC6450709

[B71] World Health Organization (2008). *WHO Standard Acupuncture Point Locations in the Western Pacific Region.* Manila: World Health Organization.

[B72] Wu T.-H. Tu C.-H. Chao H.-T. Li W.-C. Low I. Chuang C.-Y. (2016). Dynamic changes of functional pain connectome in women with primary dysmenorrhea. *Sci. Rep.* 6:24543. 10.1038/srep24543 PMC4835697 27089970

[B73] Yang Z. Fang F. Weng X. (2012). Recent developments in multivariate pattern analysis for functional MRI. *Neurosci. Bull.* 28 399–408. 10.1007/s12264-012-1253-3 22833038 PMC5561894

[B74] Yu S. Yang J. Yang M. Gao Y. Chen J. Ren Y. (2015). Application of acupoints and meridians for the treatment of primary dysmenorrhea: a data mining-based literature study. *Evid. Based Comp. Alter. Med.* 2015:752194. 10.1155/2015/752194 PMC4354715 25802545

[B75] Yu S.-Y. Lv Z.-T. Zhang Q. Yang S. Wu X. Hu Y.-P. (2017). Electroacupuncture is beneficial for primary dysmenorrhea: the evidence from meta-analysis of randomized controlled trials. *Evid. Based Comp. Alter. Med.* 2017:1791258. 10.1155/2017/1791258 PMC5735637 29358960

[B76] Yunus M. B. (2012). The prevalence of fibromyalgia in other chronic pain conditions. *Pain Res. Treat.* 2012:584573. 10.1155/2012/584573 PMC3236313 22191024

[B77] Zhang Q. Yu S. Wang Y. Wang M. Yang Y. Wei W. (2019). Abnormal reward system network in primary dysmenorrhea. *Mol. Pain* 15:1744806919862096. 10.1177/1744806919862096 PMC6616063 31286840

[B78] Zhang Y. Zhang H. Nierhaus T. Pach D. Witt C. M. Yi M. (2019). Default mode network as a neural substrate of acupuncture: evidence, challenges and strategy. *Front. Neurosci.* 13:100. 10.3389/fnins.2019.00100 30804749 PMC6378290

[B79] Zhao Z.-Q. (2008). Neural mechanism underlying acupuncture analgesia. *Prog. Neurobiol.* 85 355–375. 10.1016/j.pneurobio.2008.05.004 18582529

[B80] Zung W. W. (1971). A rating instrument for anxiety disorders. *Psychosomatics* 12 371–379. 10.1016/s0033-3182(71)71479-0 5172928

[B81] Zung W. W. K. Richards C. B. Short M. J. (1965). Self-rating depression scale in an outpatient clinic: further validation of the SDS. *Arch. Gen. Psychiatry* 13 508–515. 10.1001/archpsyc.1965.01730060026004 4378854

