# Supplementary material for: Resting-State Functional Connectivity Patterns Predict Acupuncture Treatment Response in Primary Dysmenorrhea
Source: Front Neurosci. 2020 Sep 8;14:559191. doi: 10.3389/fnins.2020.559191 (PMC7506136; doi:10.3389/fnins.2020.559191)
Supplement: Supplementary file 1 [file Image_1.pdf]

Supplementary Materials

Resting-state functional connectivity patterns predict acupuncture treatment response in primary dysmenorrhea

Siyi Yu[a,†], Mingguo Xie[b,†], Shuqin Liu[b], Xiaoli Guo[a], Jin Tian[a], Wei Wei[a], Qi Zhang[a], Fang Zeng[a], Fanrong Liang[a,*], Jie Yang[a,*]

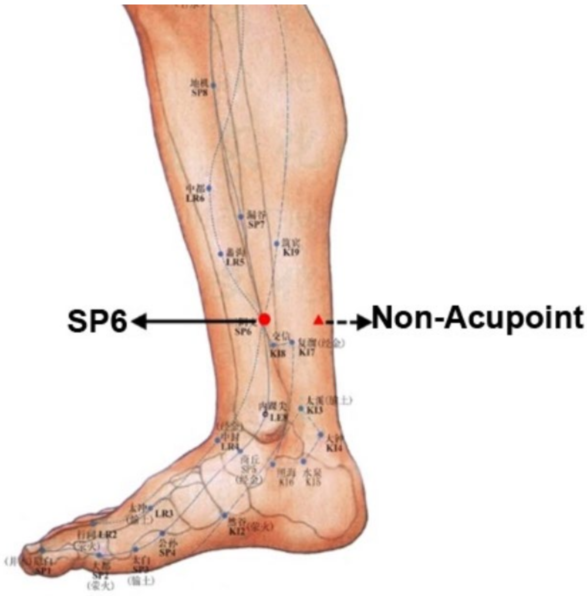

Figure S1. The acupoint selected in the present study. SP6 was selected as the target for the real acupuncture treatment, while a non-acupoint near SP6 was selected as the target for the sham acupuncture treatment.

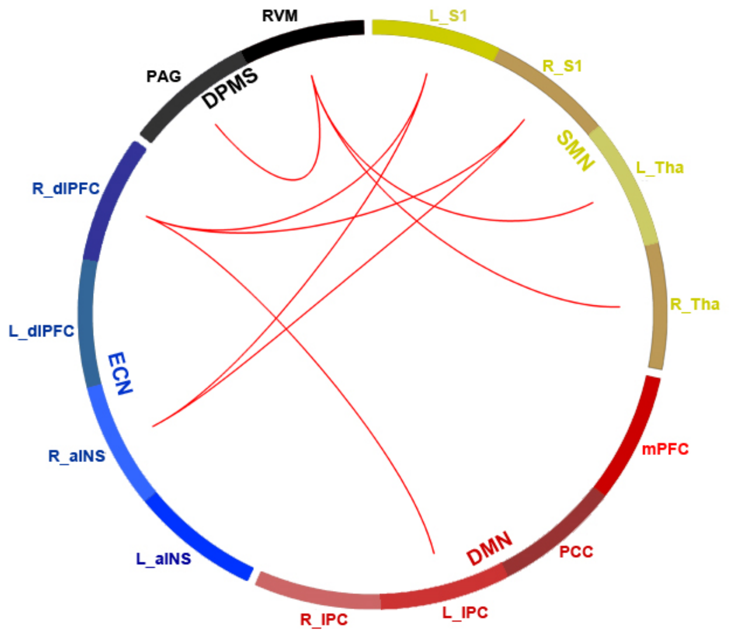

Figure S2. The different neural mechanisms altered following treatment with real and sham acupuncture for primary dysmenorrhea.
